# Capecitabine-based triplet (TPX) as induction chemotherapy in advanced head and neck cancer: a daycare-based cohort study

**DOI:** 10.3332/ecancer.2026.2124

**Published:** 2026-05-13

**Authors:** Rituparna Biswas, Krishnangshu Bhanja Choudhury, Anirban Halder

**Affiliations:** 1 Department of Radiation Oncology, Malda Medical College, Malda, West Bengal 732101, India; 2 Department of Radiation Oncology, Manipal Hospital Dhakuria, Kolkata, West Bengal 700029, India; a https://orcid.org/0000-0002-2585-3283; b https://orcid.org/0000-0002-4336-1485; c https://orcid.org/0000-0002-2721-5031

**Keywords:** induction chemotherapy, squamous cell carcinoma of the head and neck, capecitabine, feasibility studies

## Abstract

**Background:**

Head and neck squamous cell carcinoma (HNSCC) remains a global health burden. Induction chemotherapy using the regimen docetaxel, cisplatin and 5-fluorouracil (TPF) improves outcomes in advanced cases but requires continuous 5-FU infusion, limiting feasibility in daycare settings. Capecitabine, an oral prodrug of 5-FU, offers a convenient alternative. This study evaluates the efficacy and tolerability of a capecitabine-based triplet regimen docetaxel, carboplatin, capecitabine (TPX) as induction chemotherapy in a resource-limited, outpatient setting.

**Methods:**

This retrospective cohort study included 52 patients with stage IVA–IVB HNSCC treated between August 2023 and March 2025 at a Medical college in West Bengal, India. Patients were intended to receive four cycles of TPX every 21 days. Tumour response was assessed using Response Evaluation Criteria in Solid Tumours, version 1.1 after induction and following definitive therapy (surgery or chemoradiotherapy (CTRT)). Toxicities were recorded per Common Terminology Criteria for Adverse Events, version 5.0. Primary outcomes were response rates and toxicity; secondary outcome was feasibility of proceeding to curative treatment.

**Results:**

The cohort (median age 46 years; 65.4% male) had 38.5% oral cavity tumours. Following induction chemotherapy, the overall response rate (ORR) was 84.6%, with 100% ORR after definitive treatment. Oral cavity tumours showed 80% partial response (PR) post-induction; ten patients underwent successful surgery, while others received CTRT. In non-oral cavity cancers, 75% had a PR and 12.5% achieved a complete response post-induction. Toxicity was manageable; grade 3/4 neutropenia occurred in 9.6%, high-grade non-hematologic toxicity was also meagre and no treatment-related deaths were reported. Most patients (76.9%) completed all four cycles.

**Conclusion:**

The TPX regimen is a well-tolerated and effective alternative to TPF for induction chemotherapy in advanced HNSCC, especially in daycare settings. It enables high response rates and facilitates curative treatment while avoiding the logistical challenges of infusional 5-FU. Further prospective studies are warranted to confirm survival benefits and support broader adoption.

## Introduction

Head and neck squamous cell carcinoma (HNSCC) remains a significant global health challenge, accounting for over 500,000 new cases annually and ranking as the sixth most common malignancy worldwide [1]. For patients with locally advanced or unresectable disease, induction chemotherapy followed by definitive chemoradiotherapy (CTRT) or surgery has been widely utilised to improve tumour control and survival outcomes.

Among induction regimens, the combination of docetaxel, cisplatin and 5-fluorouracil (TPF) has emerged as the standard of care, primarily due to the results of two pivotal phase III trials – TAX 323 and TAX 324. These studies demonstrated significantly improved response rates, progression-free survival and overall survival in patients treated with TPF compared to platinum-fluorouracil doublets [2, 3].

However, the 5-FU component of the TPF regimen requires continuous infusion over 4–5 days, necessitating inpatient care or ambulatory infusion pumps with central venous access. This approach is impractical in centres that offer only daycare chemotherapy, as it increases logistical complexity, cost and the risk of catheter-associated complications [4].

Capecitabine, an oral fluoropyrimidine carbamate, is enzymatically converted to 5-FU primarily in tumour tissues due to elevated thymidine phosphorylase expression, leading to increased intra-tumoural drug activation and reduced systemic exposure [5, 6]. Capecitabine has been shown to offer comparable efficacy to intravenous 5-FU in gastrointestinal, breast and head and neck cancers, with the added benefits of improved convenience and a favourable toxicity profile [7–9].

Several studies have explored the use of capecitabine as a substitute for 5-FU in head and neck cancers, including in induction, concurrent and palliative settings. Combinations involving capecitabine with platinum agents and taxanes such as docetaxel have demonstrated promising response rates and manageable toxicities [10, 11]. This strategy becomes particularly relevant in settings like ours, where infrastructural limitations preclude prolonged infusional chemotherapy.

In this study, we retrospectively analysed the outcomes of Docetaxel, Carboplatin and Capecitabine (TPX) regimen comprising TPX in patients with advanced HNSCC treated in our daycare facility. Our objective was to assess the feasibility, efficacy and safety of this capecitabine-based approach in a resource-constrained setting.

## Methods

### Study design and setting

A retrospective cohort study was conducted at the Oncology department of our institution (a Medical College in West Bengal, India).

### Eligibility criteria

Patients with stage IVA (unresectable or borderline resectable or multiple large neck nodes <6 cm) to IVB (very advanced) cancer of head and neck according to the eighth edition of the American Joint Committee on Cancer (AJCC) staging system, having Eastern Cooperative Oncology Group (ECOG) performance status of 0 or 1 and bearing adequate organ function were included in the study. Age limit of 18–65 years was permitted. Key exclusion criteria included distant metastasis, a history of previous exposure of chemotherapy or radiotherapy, severe coexisting illness, non-squamous histology of cancer and pregnancy or lactation.

### Procedure

Patients fitting afore mentioned criteria are routinely subjected to induction chemotherapy with the following triple drug regimen at our department. Four 21-day cycles of TPX (intravenous Docetaxel (75 mg/m^2^, day 1), intravenous Carboplatin ((AUC = 5, day 1) and oral Capecitabine (825 mg/m^2^ orally twice daily, days 1–14)) are usually planned. As the department operates a daycare facility for chemotherapy administration, it is not equipped to support prolonged infusion regimens.

### Assessment and data collection

Primary objectives were to determine tumour response rates and toxicity profile. Patients were assessed for response via physical examination and imaging (Magnetic Resonance Imaging or Computed Tomographic scan of the face and neck using contrast) 2 weeks following the completion of neoadjuvant chemotherapy (NACT) and 6 weeks after additional treatment (in the form of Surgery or definitive Radiotherapy with o﻿r without concurrent 3-weekly Cisplatin). The same imaging modality used at baseline was repeated for response assessment. Tumour responses were evaluated based on the Response Evaluation Criteria in Solid Tumours, version 1.1 (RECIST v1.1) Table 1 [12]. Patients receiving chemotherapy are routinely assessed for treatment-related toxicities prior to each subsequent cycle. Acute toxic effects during chemotherapy were graded with the Common Terminology Criteria for Adverse Events, version 5.0 [13]. The secondary objective was to evaluate the proportion of patients who could subsequently undergo treatment with curative intent, including surgical intervention or protracted radiotherapy regimens. Only those with complete follow-up data till treatment completion, between 1 August 2023, and 31 March 2025, were included in the analysis.

## Results

### Patient characteristics

A total of 52 patients meeting the eligibility criteria during the study period were retrospectively analysed. Baseline demographic and clinical details are summarised in Table 2. The cohort was predominantly male (65.4%) with a median age of 46 years. Most patients (86.5%) had an ECOG performance status of 1. Site wise distribution is detailed in the Table 2, where 38.5% represented oral cavity cancers, while the remaining 32 patients (61.5%) had tumours originating from other head and neck subsites.

### Treatment outcomes

Response rates following NACT and completion of definitive treatment are detailed in Table 3. The overall response rate (ORR) following triplet chemotherapy was encouraging, with 84.6% of patients achieving at least a partial response (PR). Notably, all patients demonstrated either a complete response (CR) or PR by the end of treatment (ORR = 100%).

Given the known lower chemosensitivity of oral cavity tumours [14], subgroup analyses were performed. Among patients with oral cavity cancers (*n* = 20), 16 (80%) achieved PR following NACT, while two patients had stable disease (SD) and two progressed (PD). None achieved CR at this stage. Of the 16 patients with PR, 10 were deemed resectable and underwent successful margin-negative surgery. The remaining six, along with the four patients with SD or PD, received definitive CTRT. Post-treatment assessments revealed CR in five and PR in five. Patients with residual disease were subsequently managed with oral metronomic chemotherapy (OMCT).

In the non-oral cavity subgroup (*n* = 32), response to NACT included 24 patients (75%) with PR, four with SD and four who achieved CR. No cases of progression were recorded. However, four patients defaulted from further treatment – two with CR and two with PR. Of the remaining 28 patients, all except two received definitive radiotherapy with concurrent weekly cisplatin (40 mg/m²). The exceptions, who had achieved CR with induction chemotherapy, declined concurrent chemotherapy. Among these 28, 21 (75%) achieved CR and seven had PR; the latter group was subsequently treated with OMCT.

### Treatment tolerability and toxicity

Toxicity data are summarised in Table 4. No treatment-related mortality was observed. The incidence of high-grade (Grade 3/4) toxicities was low for both haematological and non-haematological events. Supportive care protocols included the use of emollients to prevent capecitabine-induced hand–foot syndrome (HFS), disinfectant mouthwashes for mucositis, prophylactic filgrastim for neutropenia, multivitamin supplementation including pyridoxine and anti-emetic prophylaxis including NK1 receptor antagonist and steroids.

Although four cycles of NACT were planned for all patients, only 40 completed the intended regimen. Two patients with oral cancers experienced early disease progression and discontinued after two cycles. The remaining ten patients received only three cycles, primarily due to toxicity or patient refusal. Reasons for early cessation included persistent thrombocytopenia (*n* = 3), Grade 3 diarrhoea (*n* = 1), transfusion-requiring anaemia (*n* = 2), Grade 3/4 mucositis (*n* = 2) and satisfactory tumour response with patient reluctance for further cycles (*n* = 2).

## Discussion

This study demonstrates the feasibility and efficacy of a capecitabine-based triplet regimen (TPX) as induction chemotherapy in patients with advanced HNSCC treated in a resource-limited, daycare-based setting. The observed ORR of 84.6% post-induction and 100% after definitive therapy underscores the potential of this regimen as a practical and effective alternative to standard TPF therapy.

Historically, the TPF regimen established in the TAX 323 and TAX 324 trials has demonstrated superiority over PF doublets in terms of response and survival outcomes, but its toxicity and logistical demands limit its widespread use in outpatient settings [2, 3]. Continuous infusion of 5-FU requires central venous access and prolonged hospitalisation, posing risks such as catheter-related infections, increased costs and reduced quality of life [4]. In contrast, capecitabine – a prodrug of 5-FU – offers oral administration and preferential activation in tumour tissues through thymidine phosphorylase, reducing systemic toxicity and enhancing convenience [1, 4–6].

Historically, the TPF regimen established in the TAX 323 and TAX 324 trials has demonstrated superiority over PF doublets in terms of response and survival outcomes, but its toxicity and logistical demands limit its widespread use in outpatient settings [2, 3]. Continuous infusion of 5-FU requires central venous access and prolonged hospitalisation, posing risks such as catheter-related infections, increased costs and reduced quality of life [4]. In contrast, capecitabine – a prodrug of 5-FU – offers oral administration and preferential activation in tumour tissues through thymidine phosphorylase, reducing systemic toxicity and enhancing convenience [1, 4–6].

Our findings are supported by prior studies that explored capecitabine as a substitute for 5-FU in HNSCC. Kattan *et al* [11] evaluated TPX in the recurrent/metastatic setting and reported a 43% response rate with manageable toxicity, emphasising its tolerability. Similarly, Saragiotto *et al* [10] used the same combination followed by CTRT and documented an 88% response rate, with a median follow-up of 4.6 years, confirming its long-term feasibility. In the nasopharyngeal carcinoma context, Li *et al* [15] reported significantly improved failure-free survival with a triplet regimen including capecitabine (TPX) over the PF doublet, reinforcing capecitabine’s efficacy in induction settings without added toxicity.

In our cohort, oral cavity cancers – traditionally considered less chemo sensitive – demonstrated an ORR of 80% post-induction and 100% post-definitive treatment. These findings align with Cheng *et al* [14] who suggested lower chemosensitivity in oral squamous cell carcinomas may be attributed to intrinsic mechanisms like enhanced DNA repair and resistance to apoptosis. However, the TPX regimen appears sufficiently active when integrated into a multimodal strategy, combining surgery or radiotherapy to achieve tumour control.

Non-oral cavity tumours showed higher CR rates post-definitive therapy (75%), supporting observations that oropharyngeal and laryngeal tumours respond more favourably to systemic therapy [2, 3]. Moreover, the relatively low toxicity of TPX observed in our study – with only 9.6% experiencing Grade 3/4 neutropenia and no treatment-related deaths –is in line with previously reported tolerability data for capecitabine-based regimens [1,10].

Capecitabine also offers pharmacoeconomic and logistical advantages in outpatient oncology. As emphasised by Twelves *et al* [5] patients preferred oral chemotherapy over infusion-based regimens, with improved quality of life and reduced healthcare resource utilisation. This becomes especially relevant in centres with limited infrastructure, where continuous 5-FU infusion is not feasible.

Looking forward, personalised approaches based on biomarkers like DPD deficiency and TP/DPD ratios may help optimise capecitabine use by predicting both efficacy and toxicity risk [1, 6]. Integration of such ‘precision chemotherapy’ strategies, along with ongoing trials evaluating novel combinations (e.g., capecitabine with lapatinib or vorinostat), may further expand capecitabine’s role in HNSCC management [1, 6].

Limitations of this study include its retrospective nature and modest sample size, precluding long-term survival analysis. Nonetheless, our real-world data support capecitabine’s substitution for 5-FU, particularly in induction settings where continuous infusions are not viable.

## Conclusion

This study demonstrates that the TPX regimen – combining TPX – is a feasible, effective and well-tolerated induction chemotherapy option for patients with locally advanced HNSCC, particularly in outpatient or resource-limited settings where continuous 5-FU infusion is impractical. The regimen achieved high ORRs with minimal high-grade toxicity, allowing most patients to proceed to definitive curative treatments. Despite the traditionally lower chemosensitivity of oral cavity tumours, favourable outcomes were observed when TPX was incorporated into a multimodal approach. These findings support the use of capecitabine as a practical substitute for 5-FU, offering logistical, economic and patient-centred advantages. Prospective studies with larger cohorts and survival endpoints are warranted to further validate its role and to explore personalised strategies for optimising its use.

## Conflicts of interest

The authors declare that they have no competing interests.

## Funding

Not applicable.

## Consent for publication

Written informed consent has been taken from patients/relatives.

## Author contributions

RB and KBC contributed to the conception of the study, data collection and evaluation. SKC and AH prepared the manuscript. RB, KBC, SKC and AH substantively revised it. All authors have read and approved the manuscript.

## Figures and Tables

**Table 1.** RECIST v1.1 definitions of tumour responses.

CR	Disappearance of all target lesions. Any pathological lymph nodes must have reduction in short axis to <10 mm.
PR	At least a 30% decrease in the sum of the diameters of target lesions, taking as reference the baseline sum diameters.
SD	Neither sufficient shrinkage to qualify for PR nor sufficient increase to qualify for PD. Essentially, disease is neither improving nor worsening significantly.
PD	At least a 20% increase in the sum of diameters of target lesions, taking as reference the smallest sum recorded during the study. In addition to the relative increase, the sum must also demonstrate an absolute increase of at least 5 mm. The appearance of one or more new lesions is also considered progression.

**Table 2.** Baseline demographic and clinical characteristics.

Characteristics	N = 52
Median age in years	46 (Range = 20–65years)
Sex	
Male	34 (65.4%)
Female	18 (34.6%)
Stage of cancer (AJCC 8th edition)	
IVA	21 (40.4%)
IVB	31 (59.6%)
Types of cancer	
Oral cavity cancer	20 (38.5%)
Cancer of Larynx	11 (21.2%)
Oropharynx	8 (15.4%)
Hypopharynx	9 (17.3%)
Nasopharynx	2 (3.8%)
Neck node metastases from unknown primary	2 (3.8%)
ECOG	
0	7 (13.5%)
1	45 (86.5%)
Abbreviations: AJCC- American Joint Committee on Cancer, ECOG- Eastern Cooperative Oncology Group performance status	

**Table 3.** Response rates.

Endpoint	After induction chemotherapy (n = 52)]	After additional treatment (Surgery or Radiotherapy) (n = 48)
CR	4 (7.7%)	36 (75%)
PR	40 (77%)	12 (25%)
SD	6 (11.5%)	0
PD	2 (3.8%)	0
ORR (CR +PR)	44 (84.6%)	48 (100%)
**Subset analysis for oral cavity cancers (*n* = 20)**		
**Endpoint**	**After induction chemotherapy (*n* = 20)**	**After additional treatment (Surgery or RT/ CTRT) (*n* = 20)**
CR	0	15 (75%)
PR	16 (80%)	5 (25%)
SD	2 (10%)	0
PD	2 (10%)	0
ORR (CR +PR)	16 (80%)	20 (100%)
**Subset analysis for rest of head & neck cancers (*n* = 32)**		
**Endpoint**	**After induction chemotherapy (*n* = 32)**	**After additional treatment (RT/CTRT) (*n* = 28)**
CR	4 (12.5%)	21 (75%)
PR	24 (75%)	7 (75%)
SD	4 (12.5%)	0
PD	0	0
ORR (CR +PR)	28 (87.5%)	28 (100%)
Abbreviations: RT/ CTRT = Radical Radiotherapy/ Concurrent chemoradiation. CR= Complete Response, PR= Partial Response, SD= Stable disease, PD= Progressive disease, ORR= Overall response rate		

**Table 4.** Chemotherapy associated acute adverse events.

Adverse event	Patients, No. (%) (n = 52)		
	Grade 0/1	Grade 2	Grade ¾
Acute hematologic toxicity			
Neutropenia	42 (80.8%)	5 (9.6%)	5 (9.6%)
Anemia	36 (69.2%)	11 (21.2%)	5 (9.6%)
Thrombocytopenia	44 (84.6%)	8 (15.4%)	0
Acute non- hematologic toxicity			
Oral mucositis	42 (80.8%)	8 (15.4%)	2 (3.8%)
Diarrhoea	47 (90.4%)	4 (7.7%)	1 (1.9%)
HFS	32 (61.6%)	18 (34.6%)	2 (3.8%)
Vomiting	46 (40.5%)	6 (11.5%)	0
